# Cumulative burden of psychiatric disorders and self-harm across 26 adult cancers

**DOI:** 10.1038/s41591-022-01740-3

**Published:** 2022-03-28

**Authors:** Wai Hoong Chang, Alvina G. Lai

**Affiliations:** Institute of Health Informatics, University College London, London, UK

**Keywords:** Psychiatric disorders, Cancer

## Abstract

Cancer is a life-altering event causing considerable psychological distress. However, information on the total burden of psychiatric disorders across all common adult cancers and therapy exposures has remained scarce. Here, we estimated the risk of self-harm after incident psychiatric disorder diagnosis in patients with cancer and the risk of unnatural deaths after self-harm in 459,542 individuals. Depression was the most common psychiatric disorder in patients with cancer. Patients who received chemotherapy, radiotherapy and surgery had the highest cumulative burden of psychiatric disorders. Patients treated with alkylating agent chemotherapeutics had the highest burden of psychiatric disorders, whereas those treated with kinase inhibitors had the lowest burden. All mental illnesses were associated with an increased risk of subsequent self-harm, where the highest risk was observed within 12 months of the mental illness diagnosis. Patients who harmed themselves were 6.8 times more likely to die of unnatural causes of death compared with controls within 12 months of self-harm (hazard ratio (HR), 6.8; 95% confidence interval (CI), 4.3–10.7). The risk of unnatural death after 12 months was markedly lower (HR, 2.0; 95% CI, 1.5–2.7). We provide an extensive knowledge base to help inform collaborative cancer-psychiatric care initiatives by prioritizing patients who are most at risk.

## Abstract (web summary)

A large-scale population analysis quantifies the burden of mental illness and self-harm events in patients diagnosed with the most common adult cancers and highlights opportunities for advancing patient care.

## Main

Mental illness is commonly associated with an increased risk of mortality in patients with cancer^1–3^. Patients with cancer may experience substantial psychological distress due to neuropsychiatric effects exerted by tumors, adverse reactions to physically demanding cancer treatment and substantial social and emotional impact (i.e., altered facial appearances) from cancer and its sequelae^4^. Cancer leaves permanent pathological alterations that imprint on people’s lives even when signs of active disease are no longer present. These effects, compounded by residual disability and periods of inability to work, could lead to social issues that serve to magnify psychological distress. Yet, cancer management often overshadows the recognition and treatment of psychiatric disorders. Patients with preexisting mental health conditions may be prone to relapse during their cancer journey, whereas individuals without a history of mental health may face competing demands from their cancer that could distract physicians from recognizing and diagnosing psychiatric disorders. Studies have also shown that individuals are often reluctant to seek professional mental health care^5^; of 24% of patients diagnosed with moderate or severe psychiatric disorder, only 8% had ever sought professional help^6^. Notable barriers to mental health help-seeking include a preference for self-reliance, low perceived need and negative experience or dissatisfaction with previous health-care encounters^5^. Individuals may also be worried about being labeled as mentally ill and may experience self-blame, which further prompts catastrophic emotional and behavioral reactions against themselves (self-harm or suicide)^7,8^.

Systematic evidence of the total burden of psychiatric disorders, self-harm episodes and risk of suicide and unnatural deaths is essential to aid early identification and intervention of mental illness and suicidal thoughts. Detailed evidence is, however, lacking in this area. Real-world linked electronic health record (EHR) data are uniquely well suited to address this question, because (1) the data capture psychiatric and self-harm events in both community care and inpatient settings, (2) linkage to the cancer registry provides patient-level data on all cancer diagnoses and treatment regimens and (3) linkage to the death registry allows the complete ascertainment of cause of death. Our study seeks to (1) estimate the variations in cumulative burden of five psychiatric disorders across 26 adult cancers stratified by treatment modalities and chemotherapy type, (2) estimate temporal variations in the first diagnosis of psychiatric disorder in relation to the time of the first self-harm event (prevalence ratios are calculated), (3) estimate the total burden of incident self-harm events (including recurrent events) after diagnosis of psychiatric disorder, (4) examine the subsequent risk of self-harm associated with each psychiatric disorder at different time frames, (5) examine mortality risk and excess years of life lost (YLL) comparing patients with and without incident psychiatric disorder diagnosis and (6) investigate the risk of natural and unnatural deaths following self-harm. Our study identifies the time frame and risk factors that can be used by physicians and family members to better monitor the mental health of patients with cancer. Psychiatric disorders are treatable and modifiable risk factors, and efforts to recognize, diagnose and treat these conditions could positively affect the quality of life after cancer.

## Results

### Cumulative burden of psychiatric disorders across 26 cancers

**Fig. 1:**
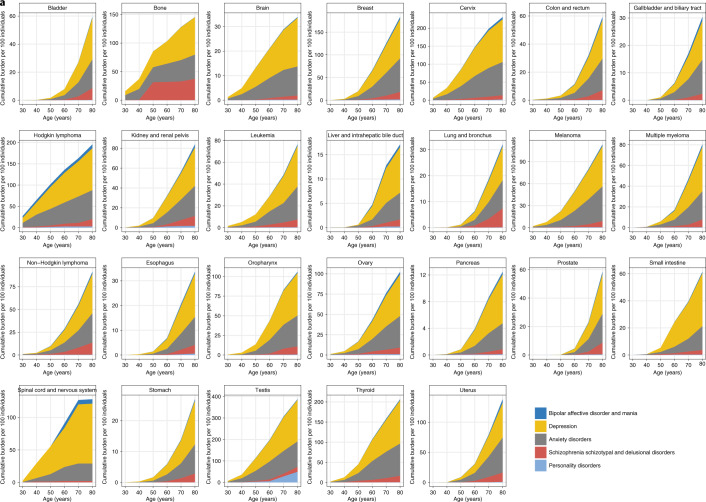

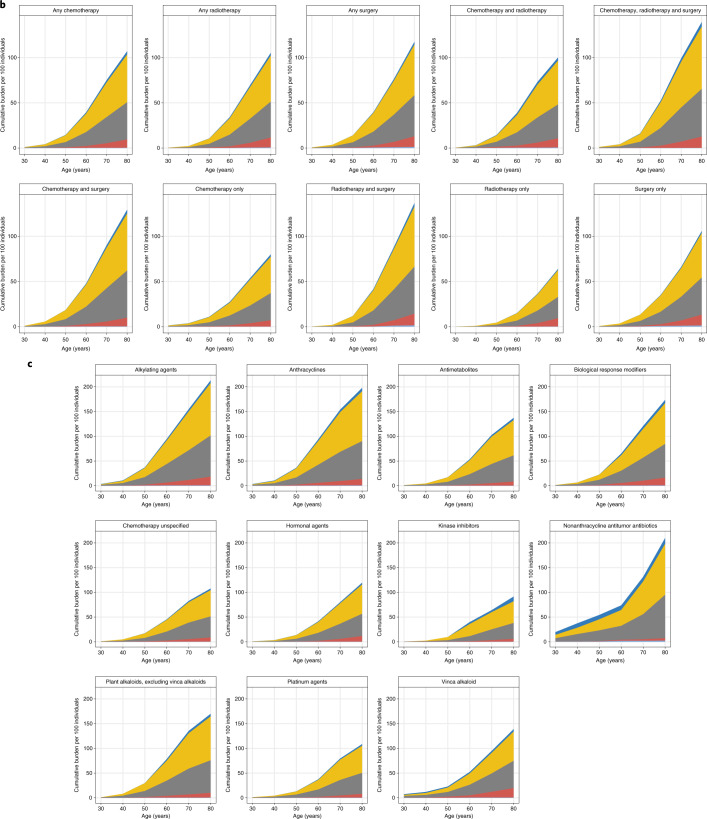
Cumulative burden of psychiatric disorders in patients with cancer. **a**–**c**, Distribution of cumulative burden by 26 cancer diagnostic groups (**a**), 10 cancer treatment modalities (**b**) and 11 chemotherapy drug classes (**c**). All data and 95% CIs are provided in the supplementary tables.

Between 1998 and 2020, we identified 459,542 individuals age ≥18 years with an incident diagnosis of a site-specific cancer of interest. Patient characteristics are presented in Supplementary Table 1. We analyzed cumulative burden of five psychiatric disorders (depression, anxiety disorders, schizophrenia, bipolar disorders and personality disorders) following cancer diagnosis and compared results across 26 cancer diagnostic groups. Cumulative burden was the highest for depression, followed by anxiety disorders (Fig. 1a and Supplementary Table 2). At age 60 years, for example, cumulative burdens per 100 individuals for depression ranked from highest to lowest by cancer type were as follows: testis, 98.05 (95% CI is used throughout, 83.08–113.02); cervix, 78.74 (73.61–83.87); Hodgkin lymphoma, 69.87 (61.05–78.69); spinal cord and nervous system, 60.37 (33.8–86.94); thyroid, 52.69 (40.74–64.64); bone, 39.17 (38.06–40.28); breast, 34.35 (33.77–34.93); melanoma, 25.98 (25.02–26.94); oropharynx, 24 (21.40–26.60); ovary, 21.92 (19.96–23.88); small intestine, 18.08 (17.47–18.69); kidney and renal pelvis, 16.24 (13.51–18.97); uterus, 14.44 (13.36–15.52); non-Hodgkin lymphoma, 13.93 (12.64–15.22); leukemia, 13.84 (12.03–15.65); brain, 11.93 (10.8–13.06); multiple myeloma, 8.5 (6.4–10.6); colon and rectum, 5.7 (5.35–6.05); bladder, 4.28 (4.19–4.37); esophagus, 3.98 (3.66-4.30); stomach, 3.2 (2.69–3.71); lung and bronchus, 3.11 (2.58–3.64); gallbladder and biliary tract, 3.04 (2.47–3.61); liver and intrahepatic bile duct, 2.75 (2.47–3.03); pancreas, 2.42 (1.94–2.90); and prostate, 2.4 (2.15–2.65) (Fig. 1a and Supplementary Table 2). Patients with testicular cancer had the highest burden across all psychiatric disorders; cumulative burdens at age 60 years were as follows: depression, 98.05 per 100 individuals (83.08–113.02); anxiety, 83.54 (78.03–89.05); schizophrenia, 8.24 (4.72–11.76); personality disorders, 5.42 (0.20–10.64); and bipolar disorders, 2.50 (1.59–3.41).

### Cancer treatment and the burden of psychiatric disorders

When comparing across treatment modalities, patients who received all three modalities (chemotherapy, radiotherapy and surgery) had the highest cumulative burden of psychiatric disorders (Fig. 1b and Supplementary Table 3). At age 60 years, cumulative burdens were as follows: depression, 28.23 per 100 individuals (27.07–29.39); anxiety, 19.66 (19.00–20.32); schizophrenia, 2.53 (2.10–2.96); personality disorders, 0.05 (0.02–0.08); and bipolar disorders, 1.94 (1.50–2.38). At age 80 years, cumulative burdens were as follows: depression, 68.77 (67.17–70.37); anxiety, 52.70 (50.89–54.51); schizophrenia, 12.50 (11.83–13.17); personality disorders, 0.16 (0.13–0.19); and bipolar disorders, 5.18 (4.05–6.31). By contrast, the lowest burden of psychiatric disorders was observed in patients who received radiotherapy alone. At age 60 years, cumulative burdens were as follows: depression, 8.09 (7.81–8.37); anxiety, 5.82 (5.55–6.09); schizophrenia, 0.80 (0.75–0.85); personality disorders, 0.09 (0.02–0.16); and bipolar disorders, 0.29 (0.20–0.38).

### Chemotherapy agents and the burden of psychiatric disorders

Patients who received alkylating agents for chemotherapy had the highest burden of psychiatric disorders. At age 60 years, cumulative burdens in patients who were treated with alkylating agents were as follows: depression, 47.55 per 100 individuals (45.31–49.79); anxiety, 37.47 (35.47–39.47); schizophrenia, 5.76 (4.84–6.68); personality disorders, 0.73 (0.60–0.86); and bipolar disorders, 3.14 (2.41–3.87) (Fig. 1c and Supplementary Table 4). Patients who received kinase inhibitor treatment, by contrast, had the lowest burden of psychiatric disorders; cumulative burdens at age 60 years were as follows: depression, 24.46 (16.95–31.97); anxiety, 10.20 (6.85–13.55); schizophrenia, 1.14 (0.62–1.66); personality disorders, 0.14 (0.00–0.28); and bipolar disorders, 3.99 (0.05–7.93).

### Temporal variations of psychiatric disorders and self-harm

**Fig. 2:**
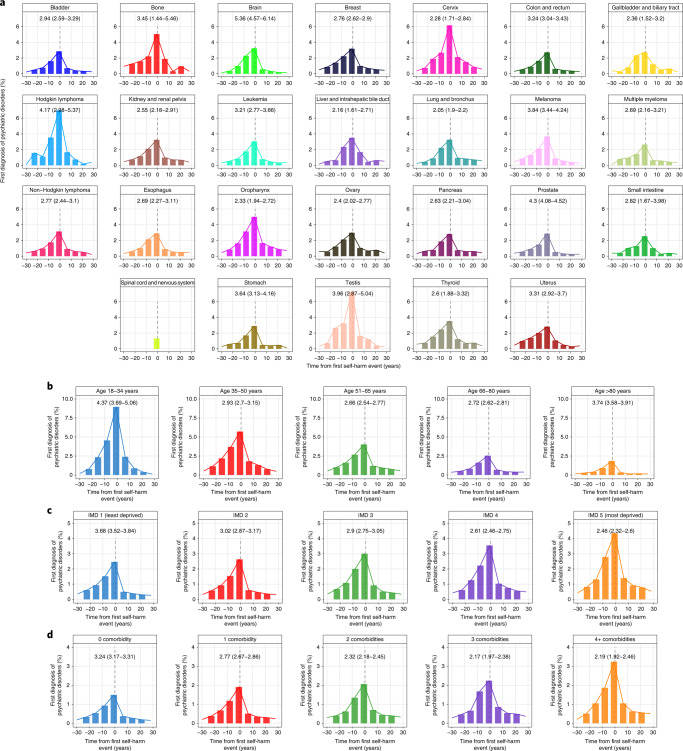
Temporal variation in the first diagnosis of psychiatric disorder in relation to the time of the first occurrence of self-harm. **a**, By cancer type. **b**, By age group. **c**, By index of multiple deprivation (IMD) status. **d**, By the number of prevalent noncancer comorbidities. Bars on the left side of the graphs indicate the proportion of individuals who were diagnosed with a psychiatric disorder before the first self-harm event. Bars on the right side of the graphs indicate the proportion of individuals who were diagnosed with a psychiatric disorder after the first self-harm event. Prevalence ratios (prevalence of psychiatric disorder diagnosis before self-harm divided by prevalence of psychiatric disorder after self-harm) are shown, alongside 95% CIs.

Among patients with cancer, 5,683 individuals were identified as having an incident self-harm episode (self-harm event after cancer diagnosis) (Extended Data Fig. 2). We observed that across cancer types, a previous diagnosis of psychiatric disorder prior to self-harm was at least twice as prevalent than a subsequent diagnosis of psychiatric disorder (Fig. 2). Prevalence ratio was the highest in patients with brain tumors (5.36; CI, 4.57–6.14), followed by prostate cancer (4.30; CI, 4.08–4.52), Hodgkin lymphoma (4.17; CI, 2.98–5.37), testicular cancer (3.96; CI, 2.87–5.04) and melanoma (3.84; CI, 3.44–4.24). By contrast, patients with lung cancer had the lowest prevalence ratio (2.05; CI, 1.90–2.20) (Fig. 2a). Younger individuals were more likely to be diagnosed with mental illness before the first self-harm episode than older individuals. For example, individuals aged 18–34 years were 4.3 times (CI, 3.69–5.06) more likely to be diagnosed with a psychiatric disorder prior to self-harm than individuals aged 51–65 years (2.66; CI, 2.54–2.77) (Fig. 2b). Patients from the most deprived regions had a lower prevalence ratio (2.46; CI, 2.32–2.60) than those from the least deprived regions (3.68; CI, 3.52–3.84), suggesting that socioeconomic deprivation had an impact on previous help-seeking behavior before self-harm (Fig. 2c). Similarly, patients with a high number of preexisting conditions were less likely to be diagnosed with a psychiatric disorder before self-harm. The prevalence ratio for individuals with 0 noncancer comorbidities was 3.24 (CI, 3.17–3.31) versus 2.19 (CI, 1.92–2.46) in individuals with four or more comorbidities (Fig. 2d).

### Self-harm after psychiatric disorder diagnosis

**Fig. 3:**
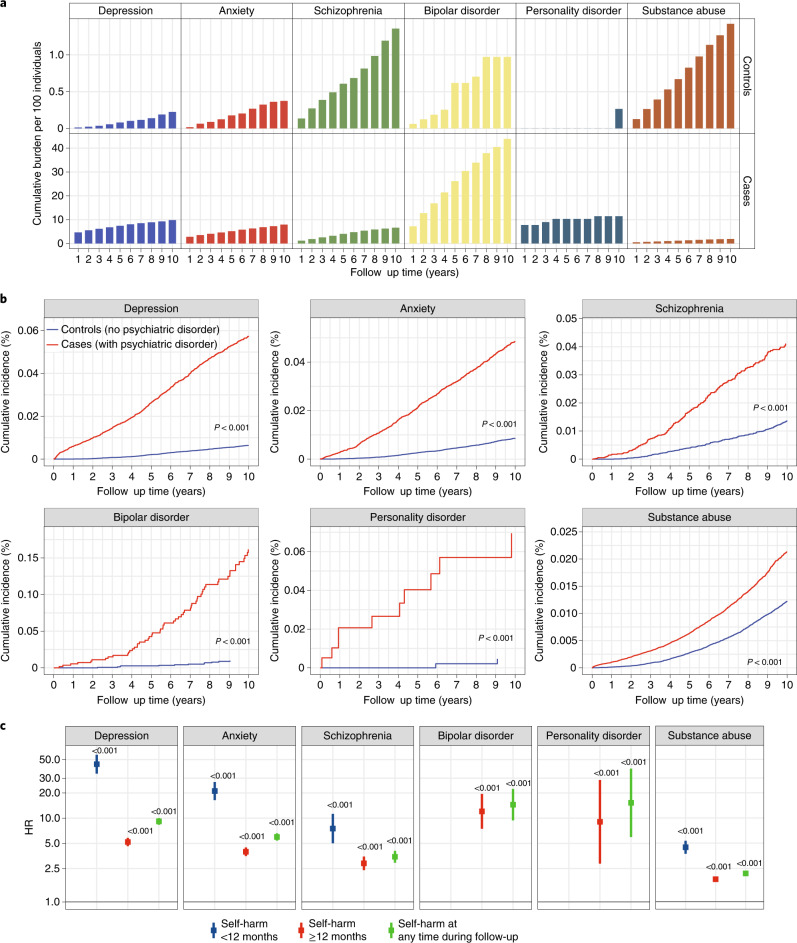
Total burden and risk of self-harm after diagnosis of psychiatric disorders in patients with cancer. Case (with psychiatric disorder) and control (no psychiatric disorder) groups were obtained via propensity score matching (Extended Data Fig. 1). Controls were matched by age at cancer diagnosis, cancer type, sex, IMD and primary care practice ID. **a**, Cumulative burden of all self-harm events in cases and controls. **b**, Cumulative incidence of the first self-harm event in cases and controls. Gray’s test was used to assess statistical significance. **c**, HRs for risk of self-harm for each psychiatric disorder were further adjusted for noncancer comorbidities, cancer treatment and presence of other psychiatric disorders. The numbers of patients with psychiatric disorder were as follows: depression, 21,609; anxiety disorder, 20,070; schizophrenia, 7,679; bipolar disorder, 557; personality disorder, 194; substance abuse, 115,868. The numbers of matched controls for each psychiatric disorder were as follows: depression, 75,087; anxiety disorder, 67,887; schizophrenia, 23,130; bipolar disorder, 1,636; personality disorder, 628; and substance abuse, 126,057. Self-harm risks during the first 12 months and subsequent years of follow-up are shown. Strata with low event numbers (*n* < 10) were not analyzed. Data are presented as HRs, and error bars represent 95% CIs. Numbers in the graphs in panel **c** represent *P* values. The likelihood ratio test was used. Full data and 95% CIs are provided in the supplementary tables.

**Extended Data Fig. 1:**
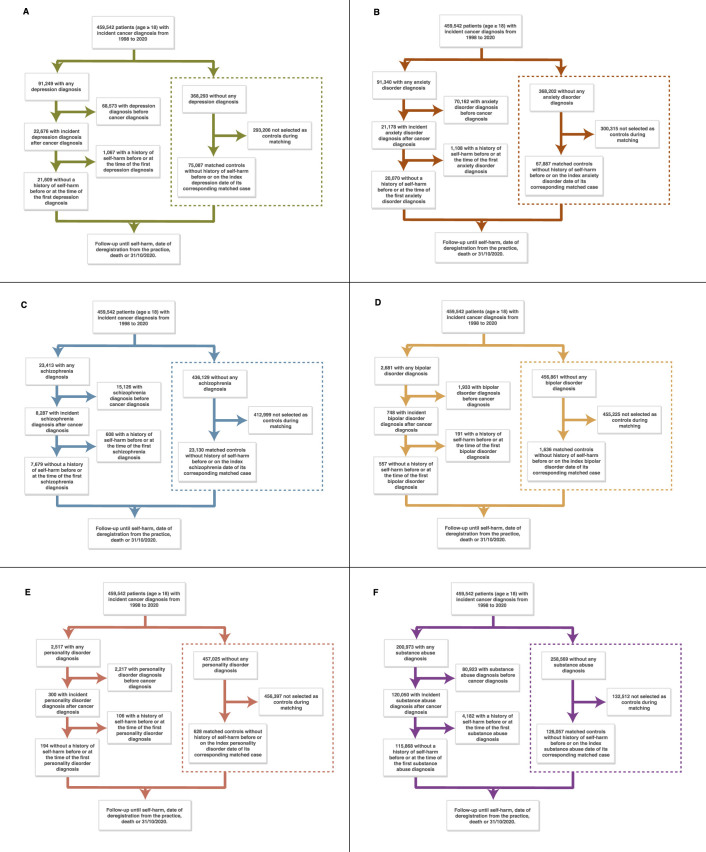
Flow diagram of case and control identification via propensity score matching for analyses on risk of self-harm after the diagnosis of psychiatric disorders in patients with cancer. Psychiatric disorders diagnostic groups are as follow: (**a**) depression, (**b**) anxiety disorders, (**c**) schizophrenia, schizotypal and delusional disorders, (**d**) bipolar affective disorder and mania (**e**) personality disorders and (**f**) substance abuse. Patients can be included in more than one diagnostic group for cases. Controls were matched by age at cancer diagnosis, cancer type, sex, Index of Multiple Deprivation and primary care practice ID.

Propensity score matching was used to identify patients with and without a specific psychiatric disorder (Extended Data Fig. 1). The numbers of patients with incident psychiatric disorders were as follows: depression, 21,609; anxiety disorder, 20,070; schizophrenia, 7,679; bipolar disorder, 557; personality disorder, 194; and substance abuse, 115,868. The numbers of matched controls for each psychiatric disorder were as follows: depression, 75,087; anxiety disorder, 67,887; schizophrenia, 23,130; bipolar disorder, 1,636; personality disorder, 628; and substance abuse, 126,057 (Extended Data Fig. 1). Patient characteristics of the six diagnostic groups for cases and controls are summarized in Supplementary Table 1. The cumulative burden of self-harm episodes was substantially higher in individuals with a prior diagnosis of psychiatric disorder compared with the controls. Across all control cohorts, the cumulative burdens of self-harm were less than 1 event per 100 controls at 10 years of follow-up, except for matched schizophrenia controls (1.36 self-harm events; CI, 1.16–1.56) and substance abuse controls (1.42 events; CI, 1.38–1.46) (Fig. 3a and Supplementary Table 5). By contrast, cumulative burden of self-harm events per 100 individuals with mental illness at 1 year of follow-up were as follows: depression, 4.65 (CI, 4.41–4.89); anxiety disorder, 2.81 (CI, 2.64–2.98); schizophrenia, 1.16 (CI, 0.95–1.37); personality disorder, 7.75 (CI, 4.65–10.85); bipolar disorder, 7.20 (CI, 5.22–9.18); and substance abuse, 0.50 (CI, 0.47–0.53). At 10 years of follow-up, the burdens of self-harm were as follows: depression, 9.83 events per 100 individuals (CI, 9.73–9.93); anxiety disorder, 7.93 (CI, 7.81–8.05); schizophrenia, 6.63 (CI, 5.92–7.43); personality disorder, 11.44 (CI, 6.91–15.97); bipolar disorder, 43.88 (CI, 40.72–47.04); and substance abuse, 1.90 (CI, 1.80–2.00) (Fig. 3a and Supplementary Table 5). Additionally, across all psychiatric disorders, the cumulative incidences for first self-harm events were consistently higher in cases than controls (Fig. 3b).

All psychiatric disorders were significantly associated with an increased risk of subsequent self-harm (Fig. 3c and Supplementary Table 6). Self-harm risk was observed to change according to the length of follow-up, with significantly higher HRs observed within 12 months of psychiatric disorder diagnosis. Individuals with depression were estimated to be 44 times more likely to self-harm during the first year (adjusted HR, 44.1; CI, 34.0–57.1). Patients with anxiety disorder or schizophrenia were 21 times (HR, 21.1; CI, 16.4–27.0) and 7 times (HR, 7.5; CI, 5.0–11.2) more likely to self-harm, respectively, during the first year. Patients with substance use disorder were 4 times (HR, 4.5; CI, 3.7–5.3) more likely to self-harm within the first year (Fig. 3c and Supplementary Table 6). The risk of self-harm markedly decreased over subsequent years of follow-up among individuals with depression (HR, 5.2; CI, 4.6–5.8), anxiety disorder (HR, 4.0; CI, 3.5–4.5), schizophrenia (HR, 2.9; CI, 2.4–3.5), bipolar disorder (HR, 12.1; CI, 7.5–19.5), personality disorder (HR, 9.0; CI, 2.9–28.5) and substance abuse (HR, 1.9; CI, 1.7–2.0) (Fig. 3c and Supplementary Table 6).

### Impact of mental illness on excess YLL

**Fig. 4:**
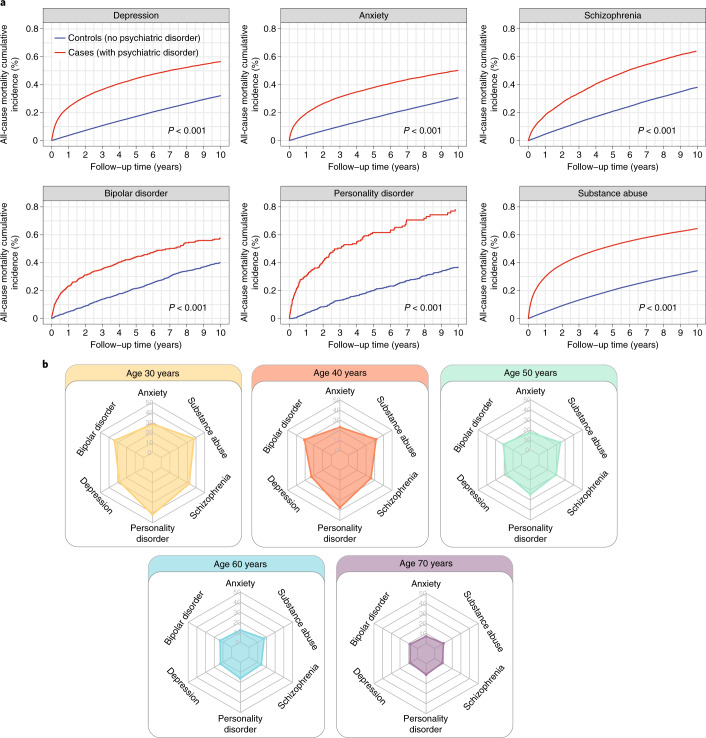
Cumulative incidence of all-cause mortality and YLL after diagnosis of psychiatric disorders in patients with cancer. Case (with psychiatric disorder) and control (no psychiatric disorder) groups were obtained via propensity score matching (Extended Data Fig. 1). Controls were matched by age at cancer diagnosis, cancer type, sex, IMD and primary care practice ID. **a**, Cumulative incidence curves for all-cause mortality after psychiatric disorder diagnosis in matched case and control groups. The log-rank test was used. **b**, Excess YLL attributable to psychiatric disorders in patients with cancer. Radar plots depict the difference in YLL between matched cases and controls. Excess YLL was estimated based on the age of onset of a psychiatric disorder. All data and 95% CIs are provided in Supplementary Table S7.

Across all psychiatric disorders, the cumulative incidences for all-cause mortality were consistently higher in cases than controls (Fig. 4a). We estimated excess YLL, which is the average number of years that patients with psychiatric disorder lose in excess of that found in patients without psychiatric disorder of the same age. Excess YLL for each psychiatric disorder across different age of onset were displayed as radar plots (Fig. 4b and Supplementary Table 7). Younger age of psychiatric disorder onset was consistently associated with higher excess YLL. At age 30 years, patients with anxiety disorder lose 28.3 years in excess of their matched controls. By comparison, excess YLL in patients diagnosed with schizophrenia at age 30 years was 32.2 years. At age 60 years, excess YLL ranked from highest to lowest were as follows:

substance abuse, 17.3 (CI, 17.2–17.4); personality disorder, 16.0 (CI, 13.8–18.2); schizophrenia, 14.1 (CI, 13.7–14.5); bipolar disorder, 13.3 (CI, 11.7–14.9); depression, 12.9 (CI, 12.6–13.2); and anxiety disorder, 12.1 (CI, 11.8–12.4) (Fig. 4b and Supplementary Table 7).

### Unnatural deaths and suicide in patients who self-harm

**Fig. 5:**
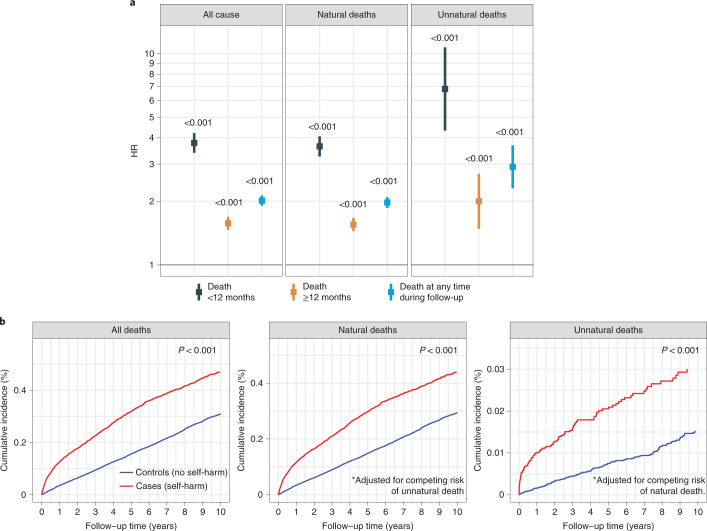
Risk of suicide and other causes of death following self-harm in patients with cancer. Case (self-harm) and control (no self-harm) groups were obtained via propensity score matching (Extended Data Fig. 2). Controls were matched by age at cancer diagnosis, cancer type, sex, IMD and primary care practice ID. **a**, HRs for risk of suicide and other causes of death were further adjusted for noncancer comorbidities, cancer treatment and presence of psychiatric disorders. Mortality risks during the first 12 months and subsequent years of follow-up are shown. We identified 5,683 individuals with incident self-harm and 18,407 matched controls. Data are presented as HRs, and error bars represent 95% CIs. The likelihood ratio test was used. Numbers in the graphs represent *P* values. **b**, Cumulative incidence curves of death due to all causes, natural causes and unnatural causes after self-harm in matched case and control groups. Gray’s test was used. *The results for natural deaths are adjusted for competing risk of unnatural deaths. The results for unnatural deaths are adjusted for competing risk of natural deaths.

**Extended Data Fig. 2:**
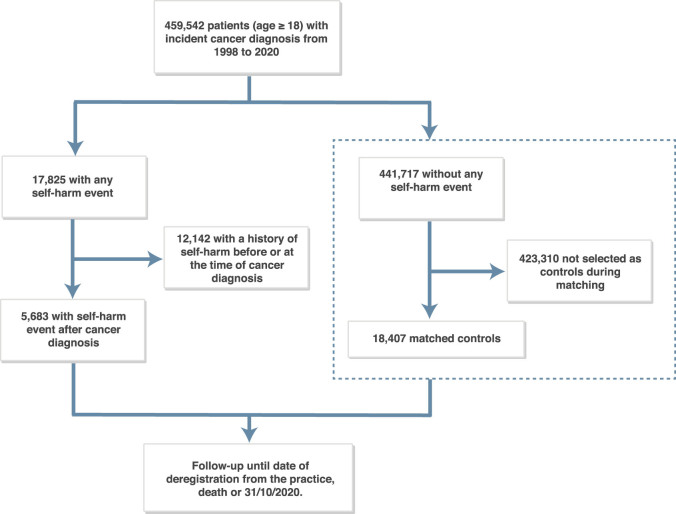
Flow diagram of case and control identification via propensity score matching for analysis on the risk of suicide and other causes of death following self-harm in patients with cancer. Controls were matched by age at cancer diagnosis, cancer type, sex, Index of Multiple Deprivation and primary care practice ID.

Propensity score matching was used to identify cases (with self-harm) and controls (without self-harm) for analyses on cause-specific mortality risk following self-harm. We identified 5,683 individuals with incident self-harm episodes and 18,407 matched controls (Extended Data Fig. 2). Adults who harmed themselves were 6.8 times more likely to die of unnatural causes of death than controls within 12 months of self-harm (HR, 6.8; CI, 4.3–10.7). The risk of unnatural death after 12 months was markedly lower (HR, 2.0; CI, 1.5–2.7) (Fig. 5a and Supplementary Table 8). Additionally, we analyzed suicides alone and found that individuals who self-harm were 25 times more likely to die of suicide (HR, 25.7; CI, 10.0–66.2). Cumulative incidence curves also demonstrated that the self-harm group had an increased risk of dying from unnatural deaths, after adjusting for competing risk of natural deaths (Fig. 5b).

## Discussion

We present the first study examining mental illness and self-harm events across 26 cancer types. Using data from primary care practices and hospitals, we quantified the total burden (not just the first event) of psychiatric disorder and self-harm. We also examined the prevalence of mental health diagnoses before and after self-harm and demonstrate that previous diagnoses of psychiatric disorders are important predictors of self-harm. We observed that prevalence ratio (prevalence of psychiatric disorder diagnosed before self-harm divided by prevalence of psychiatric disorder after self-harm) is higher among younger individuals. This difference could reflect missed diagnoses of depression in older individuals, where a self-harm event itself may trigger a psychiatric assessment that led to depression diagnosis. Our results are corroborated by another study that found that younger patients were more likely to be referred for specialized psychosocial cancer care^9^. We observed considerable differences in the risk of self-harm across psychiatric disorders. Patients with depression had the highest risk of self-harm, especially within 12 months of diagnosis, suggesting that patients require higher vigilance during this initial critical period. Interestingly, we found that schizophrenia was associated with a lower risk of self-harm, a finding that is corroborated by another study conducted in Hong Kong^10^. Patients with mental illness were significantly more likely to experience premature mortality. Furthermore, the risk of suicide and other causes of death was significantly higher in patients who harm themselves, particularly within 12 months of the first self-harm episode.

Two models have been proposed as contributing to the underlying cause of psychiatric disorders in patients with cancer: the biopsychosocial model and the neuropsychiatric effects of cancer and cancer treatment model^4^. The biopsychosocial model posits that biological, psychological and social factors contribute to chronic periods of psychological stress. Patients experiencing chemotherapy-induced alopecia are more likely to experience depression due to poorer body image and psychosocial well-being^11^. Patients may also experience health anxiety and fear of cancer recurrence, which can be triggered by internal (e.g., physical symptoms) and external cues (e.g., medical consultations, regrets about treatment decisions and media exposure)^12^. Coping with a life-threatening diagnosis of cancer may reinforce abnormal behaviors such as antisocial, narcissistic and obsessive-compulsive tendencies that define personality disorders^13^.

We observed that the cumulative burden was the highest for depression across all cancer types. For example, we demonstrated that testicular cancer patients had 98 events per 100 individuals and cervical cancer patients had 78 events followed by 69 events in Hodgkin lymphoma. A meta-analysis found that the pooled mean prevalence of depression in patients with cancer ranged from 8% to 24%, which varies across cancer types and cancer treatments^14^. Similarly, another study demonstrated that 23% and 19% of patients with cancer experienced depression and anxiety, respectively, where depression was more prevalent in patients in inpatient settings^15^. Patients with pancreatic cancer had higher levels of interleukin-6 cytokines, which is correlated with the severity of depressive symptoms^16^, but interestingly not with other measures of psychological distress. Patients with cancer may experience paraneoplastic neurologic syndromes, which are caused by immunological reactions to tumors. Paraneoplastic syndrome may induce psychiatric changes such as depression, personality disturbances, hallucinations and psychosis^17^. Patients experiencing paraneoplastic cerebellar degeneration exhibit psychiatric symptoms; for example, patients with lung cancer and limbic encephalitis present with anxiety and depression^18^.

We observed a relatively low burden of bipolar disorder in patients with cancer. Bipolar disorder typically presents at younger ages, but recent studies found that 10% of older individuals develop the first onset of manic episodes later in life^19^. Patients with bipolar disorder experience recurring manic and depressive episodes, which can be exacerbated by cancer or its treatment. Although mania is less commonly seen in patients with cancer, it may be precipitated by steroids used as part of cancer treatment^20^ or mania secondary to brain cancer. Glucocorticoid steroid-induced manic and hypomanic symptoms are common, and symptoms are thought to be dose dependent^21^. Another case report found that the chemotherapy 5-fluorouracil induced manic episodes in a patient without a history of psychiatric illness^22^. Because 5-fluorouracil can penetrate the blood–brain barrier, it is linked to neurotoxicity^23^, and mania may be caused by injury to neurotransmitter pathways. There have been limited studies on mania caused by extracerebral cancer, and neuropsychiatric symptoms reported in these situations are triggered by either steroids or paraneoplastic syndromes due to antineuronal antibodies^24^. Two reports described first-onset mania in lung cancer, and another demonstrated recurring mania in a patient with lung adenocarcinoma and a previous diagnosis of bipolar disorder^25,26^.

We observed a high burden of psychiatric disorders in patients with testicular cancer. Our results were corroborated by a recent study in Canada, where the researchers analyzed 2,619 cases of testicular cancer and found that patients with testicular cancer were more likely to have outpatient visits for mental health reasons (adjusted rate ratio, 2.45) in the peritreatment and post-treatment periods (adjusted rate ratio, 1.30) (ref. ^27^). Notably, the difference in mental health service use between testicular cancer survivors and controls lasted over 12 years. A recent systematic review evaluating the psychological distress in testicular cancer survivors concluded that testicular cancer survivors experienced significantly higher levels of anxiety (one in five survivors) and distress (one in seven survivors)^28^. One in three testicular cancer survivors experienced elevated fear or recurrence and this sense of vulnerability may have contributed to psychological distress. Men continue to experience high levels of fear of recurrence more than 10 years after the initial cancer treatment period^29^. Nonetheless, the authors cautioned against several limitations of the studies that contributed to the systematic review. Most of the studies were cross-sectional, had small sample sizes and performed only univariate analysis. This suggests that longitudinal studies on population cohorts with long-term follow-up data are still required to investigate changes over time. Another study demonstrated that patients with testicular cancer treated with chemotherapy are at greater risk of lower cognitive performance^30^, which could be related to anxiety and depression^31^. A Norwegian study on 1,408 testicular cancer survivors found that anxiety disorder was prevalent in this population, with a relative risk of 1.49. Anxiety disorder was found to be associated with young age, alcohol issues, sexual problems and peripheral neuropathy^32^.

We have shown that psychiatric disorders have a major impact on life after cancer diagnosis, where patients with psychiatric illnesses had a higher incidence of mortality and suicide risk and experienced excess YLL. Patients with schizophrenia are more likely to receive palliative care and experience premature mortality^33^, suggesting that disparities in health and cancer care exist and are influenced by the pervasive stigma of mental illness. High-intensity cancer care is associated with an increased risk of psychomotor agitation, paranoid delusions and recurrence of psychotic symptoms^34^. There has been very limited research exploring the risk of suicide and self-harm in patients with both cancer and schizophrenia. In general, patients with schizophrenia are five times more likely to commit suicide^35^. We also found that the risk of self-harm was the highest within 12 months of mental health diagnosis, suggesting that patients who are experiencing active psychological symptoms in earlier stages of the disease are more likely to harm themselves. Others have shown that the risk of suicide was 20% higher in patients with cancer compared with the general population and suggested that the first 6 months after a cancer diagnosis represent a critical period of intervention to address mental health needs^8^. A US-based study from the Surveillance, Epidemiology and End Results program found that although suicide only contributed to 0.154% of deaths in cancer patients, the risk of suicide was 4.4 times higher than that of the general population^36^. A South Korean study similarly demonstrated that patients with cancer have a higher relative risk of suicide (HR, 1.48) (ref. ^37^). Another matched case–control study found that suicide within the first year after a cancer diagnosis was significantly higher^38^. In these high-risk patients, a lower threshold for psychiatric consultation and intervention may be beneficial to reduce self-harm risk. Patients with personality disorders are often rigid and inflexible and such behaviors may affect cancer progression either through the maintenance of an unhealthy lifestyle or the inability to cope with cancer, treatments and changes in life^13^. Patients with certain personality traits can feel alienated. Neuroticism is linked to poorer quality of life after cancer treatment in patients with breast cancer^39^. Neuroticism is also associated with long-term physical (e.g., peripheral neuropathy and tinnitus) and mental (poor self-esteem and unhealthy lifestyle) morbidities in patients with testicular cancer^40^. These patients may require additional support to help them better adapt to life with cancer.

In terms of strengths, first, this is the most comprehensive study examining psychiatric disorders across 26 adult cancers, excess mortality due to mental illness, risk of self-harm and risk of suicide and other causes of deaths using a single population-based cohort. The use of population-based data means that our findings are generalizable. Second, given that we have used linked health records from primary care practices and hospitals, a particular strength is the ascertainment of psychiatric and self-harm events in ambulatory and inpatient settings. Unlike in studies based on inpatient hospital records^10^, our results are less likely to be affected by biases due to underhospitalization or underdiagnosis of psychiatric disorders. We were able to capture mild cases managed in community primary care. Third, we relied on clinically recorded diagnoses of psychiatric disorders and self-harm episodes, which means that as opposed to self-reported events, our study is free of reporting biases. Fourth, we were able to examine the effects of cancer treatment type and chemotherapy type on subsequent psychiatric events as detailed information on cancer treatment is available from the cancer registry. Fifth, linkage to the national death registry enabled the analyses on cause-specific mortality with complete case ascertainment. Sixth, our study uses the mean cumulative count (MCC) method to estimate the total burden of psychiatric and self-harm episodes over time. All other studies used the cumulative incidence method, which only considers the first event, thereby underestimating the total burden of recurring mental illness and self-harm.

We outline several limitations. We have not considered tumor stage and grade due to insufficient data. We acknowledge the possibility of surveillance bias between patients with psychiatric disorders and in those without. Although our use of population-based records provides robust and representative data, there remains a risk of underreporting of self-injurious behavior due to stigma, particularly among more affluent communities. Our analyses were adjusted for socioeconomic deprivation to reduce the impact of these biases. Suicides may be underestimated, as we have used ICD codes on death certificates as we do not have access to coroners’ reports. Coroners recording a suicide verdict must indicate suicidal intent beyond a reasonable doubt, or else an open or accidental verdict is returned^41^. To address this problem, we have included open verdicts in our analyses as recommended by others^41,42^. We have not considered treatments for psychiatric disorders. The effects of psychiatric interventions on cancer survivorship can be explored in the future.

The variations in the burden of psychiatric disorders according to cancer diagnostic category, treatment type and chemotherapy type can help inform targeted prevention strategies aimed at high-risk groups. We outline three areas for consideration: (1) early recognition and treatment of psychiatric conditions and effective monitoring after self-harm episode, (2) collaborative psychiatric and cancer care and (3) managing cancer treatment-disruptive behaviors.

Psychiatric illness may present at any point in the cancer journey. A Danish study observed an increase in incidence rates of brain and lung cancers upon the first-time psychiatric inpatient or outpatient contact. As it is not likely that the psychiatric condition itself would cause an immediate and sudden increase in cancer risk, the authors concluded that psychiatric disorders may represent one of the earliest manifestations of cancer^43^. This suggests that screening for psychiatric symptoms in cancers with paraneoplastic potential may aid in early diagnosis and treatment of both cancer and psychiatric disorder. Slow-growing cancers such as meningiomas produce psychiatric symptoms before neurological symptoms become apparent^44^. An Evidence-Based Care guideline for managing depression in patients with cancer proposed eight specific recommendations^45^: (1) screen patients with cancer for depression; (2) provide psychoeducation, destigmatize depression and investigate medical contributors to depression (i.e., vitamin B12, iron and folate levels and hypothyroidism); (3) provide pharmacologic and psychological interventions; (4) assess depression severity and follow stepped care approach; (5) consider collaborative care interventions involving oncologists, primary care practitioners and psychiatrists; (6) refer to mental health specialists when there is a risk of self-harm; (7) consider psychological therapies such as cognitive behavioral therapy; and (8) consider the use of antidepressant medication for severe depression.

Prescribing of antidepressants will need to take into account potential contraindications or drug interactions with cancer therapy. For example, the selective serotonin reuptake inhibitor antidepressant fluoxetine should be avoided in patients receiving tamoxifen treatment for breast cancer due to adverse drug interactions and increased risk of death^46^. A randomized trial (SMaRT Oncology-2) found that an integrated collaborative care model for depression in patients with cancer resulted in a better quality of life and health and reduced anxiety, pain and fatigue^47^. Patients subjected to the integrated cancer-depression care model received intensive therapy (antidepressant drugs and face-to-face psychological therapy), which highlights the importance of collaborative care approaches in achieving sustained treatment effects with a marginal increase in cost.

Disruptive behavior in patients with psychiatric illness may interfere with cancer treatment and continuing care. Unlike mental health physicians, oncologists may not receive adequate training in dealing with behavioral problems, and there has been limited guidance on managing clinical and legal risks associated with these clinically complex scenarios. Some institutes have developed policies to help physicians respond effectively to uncooperative and disruptive behavior. For example, the main principles are to focus on problem behaviors in a nonpunitive manner, introduce mental health consultation early in the cancer treatment pathway, design individualized responses to patient’s behavior and set realistic expectations of behavior^48^. Dealing with treatment-disruptive behavior can be exhausting; hence, multidisciplinary support for the primary physician is crucial, especially in the ambulatory oncology setting.

Patients with both cancer and mental illness experience premature mortality and are at greater risk of self-harm. Gaining awareness of health disparities represents an important step toward improving survival and well-being in the long-term. Our work may inform new initiatives of integrated collaborative care to identify patients who are most at risk, inform resource allocation, identify patient and institutional barriers to implementation and justify the delivery of a patient-centric model of care.

## Methods

### EHR data sources

Primary care-linked EHR databases were used. Information governance approval was obtained from the Medicines and Healthcare products Regulatory Agency (19_222). Primary care EHRs were linked to the secondary care Hospital Episode Statistics, Office for National Statistics death registry, patient-level IMD (an area-based proxy for socioeconomic deprivation) and the National Cancer Registration and Analysis Service (NCRAS). Detailed cancer registration data (cancer site, behavior, morphology and treatment information) were available in NCRAS. Chemotherapy drug details were obtained from the Systemic Anti-Cancer Treatment dataset within NCRAS, whereas radiotherapy details were obtained from the Radiotherapy dataset.

### Multiphase study designs

We identified incident primary site-specific cancer cases in individuals in England aged 18 years or older during the study period of 1 January 1998 to 31 October 2020. Incident cancers were defined as the first diagnosis of cancer occurring during the study period. First, we analyzed the cumulative burden of psychiatric disorders after cancer diagnosis. Because patients may experience recurrent psychiatric events, we used the MCC method to capture not only the first occurrence of the event but also subsequent occurrences. The cumulative burden method reflects a summarization of all events that occur in a population by a given time^49^ (see 'Cumulative burden of psychiatric disorders across 26 cancers' for details).

Second, we analyzed temporal variations in the first psychiatric disorder diagnosis in relation to the first event of self-harm. The proportion of patients being diagnosed with a psychiatric disorder (for the first time) before and after the first self-harm event was calculated and plotted. Prevalence ratios were calculated as the prevalence of psychiatric disorder diagnosis before self-harm divided by prevalence of psychiatric disorder after self-harm.

Third, we analyzed the cumulative burden of recurrent self-harm events after the diagnosis of psychiatric disorders in patients with cancer.

Fourth, we estimated the risk of self-harm after diagnosis of psychiatric disorder in patients with cancer. We identified incident cases of psychiatric disorder (patients who had a history of psychiatric disorder before cancer diagnosis were excluded) (Extended Data Fig. 1). Patients with a history of self-harm before or at the time of the first diagnosis of psychiatric disorder were also excluded. For each psychiatric disorder, the first date of diagnosis (index date) was used as the date that follow-up started. Controls were identified by propensity score matching by age at cancer diagnosis, cancer type, sex, IMD and primary care practice ID (a unique identifier for primary care practices). Matching was performed using the optimal pair matching algorithm in the R matchit package based on the premise that the sum of the absolute pairwise distances in the matched sample was as small as possible. Unlike the nearest-neighbor matching, optimal matching ensures that within-pair distances remain small. For controls, the index date of its corresponding matched case was the date that follow-up started. Patients were followed up until self-harm, date of deregistration from the practice, death or date of administrative censoring (31 October 2020), whichever occurred first (Extended Data Fig. 1).

Fifth, we estimated the risk of suicide and other causes of death following self-harm in patients with cancer. We identified incident self-harm episodes (patients who had a history of self-harm before cancer diagnosis were excluded). The first date of self-harm (index date) was used as the date that follow-up started. Controls were identified by propensity score matching by age at cancer diagnosis, cancer type, sex, IMD and primary care practice ID. For controls, the index date of its corresponding matched case was the date that follow-up started. Patients were followed up until death, date of deregistration from the practice or date of administrative censoring (31 October 2020), whichever occurred first (Extended Data Fig. 2).

### EHR phenotypes

All EHR phenotypes were obtained from an open-access phenotype library^50^ and have been previously validated^51–53^. Phenotypes for primary care were generated using version 2 Read and SNOMED CT codes. Phenotypes for Hospital Episode Statistics were generated in ICD-10. EHR phenotypes for self-harm were obtained from a previous study^42^. We used primary care, secondary care and NCRAS records to identify patients aged ≥18 years with an incident primary site-specific cancer. We considered 26 cancer types: bladder, bone, brain, breast, cervix, colon and rectum, gallbladder and biliary tract, Hodgkin lymphoma, kidney and renal pelvis, leukemia, liver and intrahepatic bile duct, lung and bronchus, melanoma, multiple myeloma, non-Hodgkin lymphoma, esophagus, oropharynx, ovary, pancreas, prostate, small intestine, spinal cord and nervous system, stomach, testis, thyroid and uterus. We considered five psychiatric diagnostic categories: depression, anxiety disorders, schizophrenia, schizotypal and delusional disorders, bipolar affective disorder and mania and personality disorders. For analyses involving matched case–control cohorts, we added substance abuse (substance use disorder).

Prevalent noncancer physical comorbidities recorded before index date entry were identified from primary and secondary care records. We considered the following 21 comorbidities: heart failure, chronic obstructive pulmonary disease, HIV, hepatic disorders (i.e., alcoholic liver disease, nonalcoholic fatty liver disease, hepatic failure, liver fibrosis and cirrhosis and portal hypertension), stroke (i.e., ischemic stroke, stroke not otherwise specified, transient ischemic attack, intracerebral hemorrhage and subarachnoid hemorrhage), myocardial infarction, vascular disease (i.e., peripheral arterial disease, Raynaud’s syndrome and venous thromboembolic disease) and abnormal glucose metabolism (i.e., type 1 or type 2 diabetes, diabetic neurological complications and diabetic ophthalmic complications). For stratified analyses, patients were divided into the following categories: no additional comorbidities (i.e., patients without diagnosis of any of the above noncancer comorbidities), one additional comorbidity, two comorbidities, three comorbidities and four or more comorbidities.

We analyzed ten cancer treatment variables: (1) all chemotherapy (i.e., everyone who received chemotherapy), (2) all radiotherapy, (3) all surgery, (4) chemotherapy only (i.e., individuals who received chemotherapy only and nothing else), (5) radiotherapy only, (6) surgery only, (7) chemotherapy and radiotherapy, (8) chemotherapy and surgery, (9) radiotherapy and surgery and (10) chemotherapy, radiotherapy and surgery. We considered 11 types of chemotherapy drug variables: (1) alkylating agents; (2) anthracyclines; (3) antimetabolites; (4) biological response modifiers, including monoclonal antibodies; (5) chemotherapy not otherwise specified; (6) hormonal agents including corticosteroid hormones and sex hormones; (7) kinase inhibitors; (8) nonanthracycline antitumour antibiotics; (9) plant alkaloids and natural products, excluding vinca alkaloids; (10) platinum agents; and (11) vinca alkaloids. Cause-specific mortality was defined using the underlying cause of death code as recorded in the Office for National Statistics death registry. Natural deaths consist of all ICD-10 codes, excluding V01-Y98. Unnatural deaths include V01-Y98 and suicides (including open verdicts X60-X84 and Y10-Y34) (ref. ^42^).

### Statistical analyses

We estimated the cumulative burden of five psychiatric disorders and self-harm episodes using the previously described and validated MCC method^49,54^. Given that psychiatric and self-harm episodes can recur, we used the MCC method, which considers recurrent events; cumulative incidence only considers the first occurrence of the event in each individual. We analyzed the burden of recurrent events in the presence of competing risks. We considered death as a competing risk event, as it precludes the occurrence of other events of interest. MCC can adopt any positive number, as it is an estimation of the mean count of events per individual within a given population (not the probability of developing the event). Cumulative burden of psychiatric disorders following cancer diagnosis was estimated as the average number of psychiatric events occurring at a given age. An MCC of 0.5 at age 40 years means that there is an average of 0.5 events occurring per individual at age 40 years (i.e., 50 events per 100 individuals). Cumulative burden of self-harm following psychiatric disorder diagnosis was estimated as the average number of self-harm events occurring at a given time of follow-up (i.e., time from psychiatric disorder diagnosis to self-harm). An MCC of 0.16 by 5 years of follow-up means that there is an average of 16 events per 100 individuals at 5 years; 95% CIs were generated using the bootstrap percentile method^49^.

We performed Cox proportional hazards regression to determine both the risk of self-harm after psychiatric disorder diagnosis and the risk of suicide and other causes of death following self-harm. Cox regression was performed on matched cohorts and further adjusted for noncancer comorbidities, cancer treatment and the presence of additional psychiatric disorders. Proportional hazards assumption was evaluated using the Schoenfeld residuals. We also estimated the cumulative incidence for all-cause mortality after psychiatric disorder diagnosis and all-cause and cause-specific mortality after self-harm. Analyses on specific causes of death were adjusted for the competing risk of dying from other causes. YLL refers to the number of years lost in patients with a given disease and was estimated using the R package lillies^55^, which was validated by other studies^56–58^. We calculated excess YLL as the average YLL (in years) that patients with psychiatric disorders experience from the time of diagnosis in excess to those experienced by the control population (i.e., individuals without psychiatric disorders) of the same age. Excess YLL was estimated based on the age of psychiatric disorder onset at ages 30, 40, 50, 60 and 70 years. Analyses were performed using R (version 3.6.3) with the following packages: tidyverse, tableone, lillies, reshape, splines, survival, survminer, etm, mstate, cmprsk, DataCombine, data.table, ggsci and pals.

### Reporting Summary

Further information on research design is available in the Nature Research Reporting Summary linked to this article.

## Online content

Any methods, additional references, Nature Research reporting summaries, source data, extended data, supplementary information, acknowledgements, peer review information; details of author contributions and competing interests; and statements of data and code availability are available at 10.1038/s41591-022-01740-3.

## Supplementary information

Reporting Summary

Supplementary Table 1. Baseline characteristics of all patients with cancer and matched case and control cohorts for each psychiatric disorder. Supplementary Table 2. Cumulative burden of psychiatric disorders stratified by cancer diagnostic groups at 30, 40, 50, 60, 70 and 80 years attained age. Cumulative burden is shown as mean number of events per 100 individuals. Supplementary Table 3. Cumulative burden of psychiatric disorders stratified by cancer treatment regimens at 30, 40, 50, 60, 70 and 80 years attained age. Cumulative burden is shown as mean number of events per 100 individuals. Supplementary Table 4. Cumulative burden of psychiatric disorders stratified by chemotherapy types at 30, 40, 50, 60, 70 and 80 years attained age. Cumulative burden is shown as mean number of events per 100 individuals. Supplementary Table 5. Cumulative burden of self-harm events in cases versus controls stratified by psychiatric disorder diagnosis. Cumulative burden is shown as mean number of events per 100 individuals. Supplementary Table 6. Multivariable Cox regression analysis for the risk of self-harm following psychiatric disorder diagnosis. Case (with psychiatric disorder) and control (no psychiatric disorder) groups were obtained via propensity score matching. Controls were matched by age at cancer diagnosis, cancer type, sex, IMD and primary care practice ID. HRs were further adjusted for noncancer comorbidities, cancer treatment and the presence of other psychiatric disorders. Supplementary Table 7. Excess YLL attributable to psychiatric disorder diagnosis in patients with cancer. Excess YLL is calculated as the difference in YLL between patients with and without psychiatric disorder diagnosis. Excess YLL was estimated based on the age of onset of a specific psychiatric disorder. Supplementary Table 8. Multivariable Cox regression analysis for the risk of suicide and other causes of death following self-harm in patients with cancer. Case (self-harm) and control (no self-harm) groups were obtained via propensity score matching. Controls were matched by age at cancer diagnosis, cancer type, sex, IMD and primary care practice ID. HRs were further adjusted for noncancer comorbidities, cancer treatment and the presence of psychiatric disorders.

## Data Availability

This study uses patient data in England collected as part of their care and support. EHR datasets included primary care, secondary care Hospital Episode Statistics, Office for National Statistics death registry, patient-level IMD and the National Cancer Registration and Analysis Service data (including Systemic Anti-Cancer Treatment and Radiotherapy datasets). Because EHRs are classified as sensitive data by the UK Data Protection Act, information governance restrictions are in place to protect patient confidentiality and prevent data sharing in public repositories. Data are available to researchers on successful ethics application to the Medicines and Healthcare products Regulatory Agency. All summarized data and results are made available as extended data items.

## Code availability

All analyses were performed using R Studio within an airlocked research environment using analysis codes retrieved from the following website: https://sites.ualberta.ca/~yyasui/software.html.
