# Supplementary material for: Cumulative burden of psychiatric disorders and self-harm across 26 adult cancers
Source: Nat Med. 2022 Mar 28;28(4):860–70. doi: 10.1038/s41591-022-01740-3 (PMC9018408; doi:10.1038/s41591-022-01740-3)
Supplement: Supplementary file 1 — Reporting Summary [file 41591_2022_1740_MOESM1_ESM.pdf]

nature portfolio

Corresponding author(s): Alvina Lai

Last updated by author(s): Feb 7, 2022

Reporting Summary

Nature Portfolio wishes to improve the reproducibility of the work that we publish. This form provides structure for consistency and transparency in reporting. For further information on Nature Portfolio policies, see our Editorial Policies and the Editorial Policy Checklist.

Statistics

For all statistical analyses, confirm that the following items are present in the figure legend, table legend, main text, or Methods section.

n/a	Confirmed	
☐	☒	The exact sample size (*n*) for each experimental group/condition, given as a discrete number and unit of measurement
☐	☒	A statement on whether measurements were taken from distinct samples or whether the same sample was measured repeatedly
☐	☒	The statistical test(s) used AND whether they are one- or two-sided<br>*Only common tests should be described solely by name; describe more complex techniques in the Methods section.*
☐	☒	A description of all covariates tested
☒	☐	A description of any assumptions or corrections, such as tests of normality and adjustment for multiple comparisons
☐	☒	A full description of the statistical parameters including central tendency (e.g. means) or other basic estimates (e.g. regression coefficient) AND variation (e.g. standard deviation) or associated estimates of uncertainty (e.g. confidence intervals)
☐	☒	For null hypothesis testing, the test statistic (e.g. *F*, *t*, *r*) with confidence intervals, effect sizes, degrees of freedom and *P* value noted<br>*Give P values as exact values whenever suitable.*
☒	☐	For Bayesian analysis, information on the choice of priors and Markov chain Monte Carlo settings
☒	☐	For hierarchical and complex designs, identification of the appropriate level for tests and full reporting of outcomes
☒	☐	Estimates of effect sizes (e.g. Cohen's *d*, Pearson's *r*), indicating how they were calculated

Our web collection on statistics for biologists contains articles on many of the points above.

Software and code

Policy information about availability of computer code

Data collection	Population electronic health records were employed.
Data analysis	Analysed were performed using R Studio (version 3.6.3) with the following packages: tidyverse, tableone, lillies, reshape, splines, survival, survminer, etm, mstate, cmprsk, DataCombine, data.table, ggsci and pals.

For manuscripts utilizing custom algorithms or software that are central to the research but not yet described in published literature, software must be made available to editors and reviewers. We strongly encourage code deposition in a community repository (e.g. GitHub). See the Nature Portfolio guidelines for submitting code & software for further information.

Data

Policy information about availability of data

All manuscripts must include a data availability statement. This statement should provide the following information, where applicable:

- Accession codes, unique identifiers, or web links for publicly available datasets
- A description of any restrictions on data availability
- For clinical datasets or third party data, please ensure that the statement adheres to our policy

This study employs patient data in England collected as part of their care and support. Electronic health record datasets include primary care, secondary care Hospital Episode Statistics, Office for National Statistics death registry, patient-level Index of Multiple Deprivation and the National Cancer Registration and Analysis Service data (including Systemic Anti-Cancer Treatment and Radiotherapy datasets). Since electronic health records are classified as sensitive data by the UK Data Protection Act, information governance restrictions are in place to protect patient confidentiality and prevent data sharing in public repositories. Data are available to researchers on successful ethics application to the Medicines and Healthcare products Regulatory Agency. All summarised data and results are made available as extended data items.

Field-specific reporting

Please select the one below that is the best fit for your research. If you are not sure, read the appropriate sections before making your selection.

☒ Life sciences ☐ Behavioural & social sciences ☐ Ecological, evolutionary & environmental sciences

For a reference copy of the document with all sections, see nature.com/documents/nr-reporting-summary-flat.pdf

Life sciences study design

All studies must disclose on these points even when the disclosure is negative.

Sample size	We analysed 459,542 individuals age ≥ 18 years with an incident diagnosis of a site-specific cancer of interest. Sample size for each analysis was determined using multiphase study designs as outlined in the Methods.
Data exclusions	Only incident primary site-specific cancer cases were included.
Replication	As this is an observational epidemiological study using a population-based cohort, experimental replication is not applicable.
Randomization	This is an observational epidemiological study. Thus randomisation is not applicable. However, we have taken measures to ensure that cases and controls were comparable using propensity score matching.
Blinding	This is an observational epidemiological study thus blinding is not applicable.

Reporting for specific materials, systems and methods

We require information from authors about some types of materials, experimental systems and methods used in many studies. Here, indicate whether each material, system or method listed is relevant to your study. If you are not sure if a list item applies to your research, read the appropriate section before selecting a response.

Materials & experimental systems

n/a	Involved in the study	
☒	☐	Antibodies
☒	☐	Eukaryotic cell lines
☒	☐	Palaeontology and archaeology
☒	☐	Animals and other organisms
☐	☒	Human research participants
☒	☐	Clinical data
☒	☐	Dual use research of concern

Methods

n/a	Involved in the study	
☒	☐	ChIP-seq
☒	☐	Flow cytometry
☒	☐	MRI-based neuroimaging

Human research participants

Policy information about studies involving human research participants

Population characteristics	We identified 459,542 individuals age ≥ 18 years with an incident diagnosis of a site-specific cancer of interest. 50% were male and the mean age of the population was 68.8 years. The number of patients with incident psychiatric disorder were as follow: depression (21,609), anxiety disorder (20,070), schizophrenia (7,679), bipolar disorder (557), personality disorder (194) and substance abuse (115,868). The number of matched controls for each psychiatric disorder were as follow: depression (75,087), anxiety disorder (67,887), schizophrenia (23,130), bipolar disorder (1,636), personality disorder (628) and substance abuse (126,057).
Recruitment	This study employs population health records from English patients that were collected as part of routine clinical care.
Ethics oversight	Ethics approval was obtained from the Medicines and Healthcare products Regulatory Agency (19_222).

Note that full information on the approval of the study protocol must also be provided in the manuscript.
